# Location-Based LTE-M Uplink Power Control and Radio Resource Scheduling

**DOI:** 10.3390/s22041474

**Published:** 2022-02-14

**Authors:** Jibao Wang, Hailin Jiang

**Affiliations:** National Engineering Research Center of Rail Transportation Operation and Control System, Beijing Jiaotong University, Beijing 100044, China; 20120251@bjtu.edu.cn

**Keywords:** LTE-M, radio resource scheduling, SFR, proportional fair

## Abstract

Long-Term Evolution for Metro (LTE-M) is adopted as the data communication system in urban rail transit to exchange bio-direction train-wayside information. Reliable data communication is essential in LTE-M systems for ensuring trains’ operation safety and efficiency. However, the inter-cell inference problem exists in LTE results in throughput reduction, especially when trains are in the edge area of adjacent cells, and has negative effects on train operation. The uplink power control and radio resource scheduling scheme is studied in LTE-M system which differentiates from public cellular networks in user numbers and the availability of the trains’ locations. Since the locations of the trains are available, the interferences from the neighbouring cells can be calculated, and a location based algorithm together with soft frequency reuse is designed. In addition, a proportional fair algorithm is taken to improve uplink radio resource scheduling considering the fairness to different train-wayside communication service requirements. Through simulation, the practicability of the proposed schemes in communication system of urban rail transit is verified in aspects of radio power control and data communication throughput.

## 1. Introduction

With the development of urban rail transit in big cities, the DCS (Data Communication System) in it also introduces advanced and reliable technologies. LTE-M (Long Term Evolution for Metro) is a train-wayside communication system for integrated service including CBTC (Communication-Based Train Control) and IMS (Image Monitoring System) in urban rail transit/metro based on TD-LTE technology [1,2]. TD-LTE is a time division duplex communication system based on OFDM (Orthogonal Frequency Division Multiplexing) technology with a frequency reuse factor of 1. Due to the orthogonality of OFDM, the signal interference within one cell is small, but the performance of the LTE-M system is influenced by inter-cell interference (ICI) because all cells adopt the same frequency band, especially when trains are in a cell-edge area. Technical specification of the LTE system defines some methods for ICI coordination, which rely on exchange of radio resource interference and load information such as OI (Overload Indicator) and HII (High Interference Indicator) between cells and the adoption of scheduling algorithms to avoid or eliminate interference. Moreover, LTE standards define a power control algorithm in the uplink direction so that the strength of the signal transmitted by different user equipment could be maintained at a stable level in the base station. However, the power-control algorithm does not clearly specify how to reduce the impact of inter-cell interference [3].

The biggest difference between public LTE and LTE-M DCS in urban rail transit is the number of users in the network. There are hundreds of users in one cell usually, but in the latter case user number is very small, which does not exceed four and is less than six even in extreme situation. Moreover, metro lines and trains’ trajectory are fixed; thus, UE (User Equipment) locations can be easily obtained and mutual interference information between cells can be calculated accurately. Considering the position of trains in metro lines, a power-control algorithm based on location is designed in this paper with interference coordination methods of SFR (Soft Frequency Reuse).

Nowadays, public LTE system and other mobile networks adopt a cellular networking scheme. However, co-channel interference problem arises and becomes serious sometimes because the same frequency resource can be used between adjacent cells, especially when the LTE system has a reuse factor of 1. Uplink radio power-control methods are used to limit the transmit power of UEs to mobile base station and keep the receiving powers at nearly the same level. The limited transmit power of UEs can reduce ICI to some extent. The common anti-interference methods can be classified into three types: interference coordination, interference cancellation and interference randomization [4]. Among them, ICI coordination schemes are relatively easy to operate, and the other two are more difficult and complex to implement. The main idea of ICI coordination is making a reasonable allocation of time and frequency radio resources between cells to ensure these allocations in edge areas are different from each other. The problem caused by the same frequency thereby can be reduced.

The interference coordination methods can be divided into static, semi-static and dynamic ways. Static interference coordination applies radio resource planning or pre-configuration methods to limit allocation strategy and resource scheduling of each cell to avoid mutual interference [5]. FFR (Fractional Frequency Reuse) and SFR (Soft Frequency Reuse) technologies are both typical static interference coordination methods for LTE system. Compared with the FFR, spectrum utilization under the SFR mode is improved clearly [6,7], but all of them are static methods at present. Since trains locations change rapidly in urban rail transit, the throughput of TAU (Train Access Unit, the mobile terminal on train) may fluctuate significantly over time and space especially when trains are in cell edge area, which is the weakest part of LTE-M systems and has the risk of network security [8,9]. Therefore, the use of static resource scheduling schemes may cause huge communication capacity loss. The dynamic frequency reuse method can improve efficiency for UEs as described in [10]. Uplink power control in LTE systems plays an important role in the mentioned ICI coordination methods. Reference [11] proposes a co-operative power control method using the utility function to improve cell edge user performance and [12] applies machine learning in power control to maximize the spectral efficiency, but those methods mentioned above are too complex and not suitable for the urban subway communication environment. The authors of [13] comprehensively introduce interference management using FFR and SFR in a hybrid network based on surrounding interferences, which are randomly created, like most public cellular network scenarios. The research about the ICI problem in LTE-M systems is limited. Zhu takes the dynamic SFR method to study this issue in urban rail transit and achieves some results in [14], but the factor of train locations is not taken into consideration. Compared with these mentioned works, this paper contributes to developing a novel power-control and scheduling algorithm based on the location of terminals in urban rail transit. Since the position of all UEs in serving cells and neighbor cells can be learned, which is different from traditional methods that suppose the interferences from adjacent cells are random, the transmit power can be calculated and scheduled according to interference limits and service requirements in a fixed manner. The simulations also verify that the methods are effective.

The rest of paper is organized as follows. LTE-M system architecture and the model used in this work are described in Section 2. Problem formulation is introduced in Section 3 and detailed location and SFR-based algorithm design for uplink power control and PF-based radio resource scheduling algorithm in the LTE-M system are presented in Section 4. Section 5 introduces simulation parameters and results analysis. Finally, the main conclusions are drawn in Section 6.

## 2. System Model

**Figure 1:**
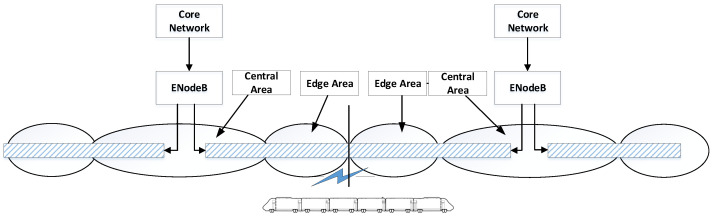
LTE-M Train-wayside Data Communication System in Urban Rail Transit.

The urban rail transit LTE-M train-wayside communication system can be simply shown as in Figure 1. Considering the tunnel coverage and co-channel interference of different systems (airports, electric power and other industries can also use a 1.8GHz frequency band like the urban rail transit system), most communication scenes are covered by leaky cables [15]. In order to avoid cumbersome tuning of handover parameters between cells, the leaky cables are connected directly. In the coverage of one cell, it is assumed that the entire area is divided into a central area and an edge area. Central area is relatively close to the base station. Terminals in this area can use a smaller transmit power to transfer signal to the uplink, and the SNR at receiver can also meet requirements. Users in the edge area are relatively far away from the base station. In order to make the received signal in one cell reach the target SNR, terminals need to transmit signals with greater power. However, because the LTE system is a co-frequency network, high transmit power of terminal in the edge area of one cell will cause serious interference to neighboring cells.

An advantage of leaky cable as a transmission medium is uniform coverage. The multipath component of wireless signals in leaky cable is much less than that of wireless free waves. The large-scale fading formula of the signal in 1.8 GHz leaky cable is described as Formula (1).

(1) $$PL\left(d\right)=PL_{c}+d\times PL_{T}$$

In this formula, $PL\left(d\right)$ is the predicted path loss when transmission distance is *d*, $PL_{c}$ is the coupling loss of the leaky cable, and $PL_{T}$ is the transmission loss coefficient of leaky cable. Therefore, by measuring the received signal strength of the terminal such as RSRP (Reference Signal Receiving Power), the distance between the train and base station can be accurately calculated. In addition, train in operation needs to send position information to the urban rail transit controller center periodically [15,16]. The period for reporting of location is 200 ms and the max speed of a subway train is about 80 km/h. Therefore, the running distance of the train in one reporting circle is about 4.4 m. The position information of terminals can be obtained more accurately in this way.

Furthermore, the number of trains in one cell in the LTE-M system usually does not exceed four, and this paper assumes that there are four trains at most in one cell at the same time. If there are no trains in the edge area, there is no need to consider interference restriction in this area when neighboring cells schedule resources. In order to implement uplink power control and interference coordination more efficiently, the information about whether there are trains or not in the edge area of cells has to be sent to neighboring cells. There are four trains in one cell and the corresponding information to be sent needs 4 bits. If the coverage area of one cell is further subdivided into *L* areas, namely $D_{1},D_{2},\cdots ,D_{L}$ according to the distance from the base station near to far, the information that needs to be transmitted between cells is 4×L bits.

In order to apply SFR technology to perform uplink radio resource scheduling, different physical time and frequency resources need to be allocated to the central area and edge area. In the LTE system, time and frequency radio resources are scheduled in units of PRB (Physical Resource Block). It is assumed that the total bandwidth of LTE-M system is 10 MHz. In urban rail transit, two identical but completely isolated LTE-M systems (called blue and red network) are required to work simultaneously to ensure reliable data communication. Therefore, the bandwidth of one LTE-M system is 5MHz, which corresponds to 25 PRBs.

**Figure 2:**
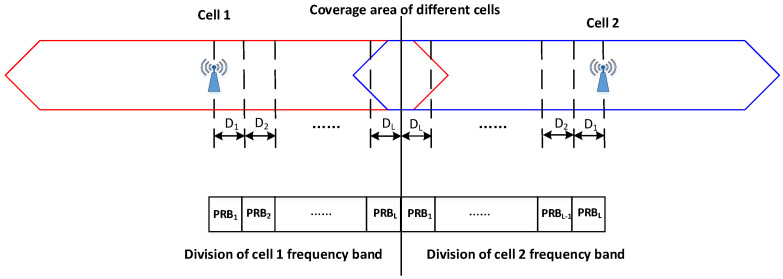
Frequency band in different coverage areas in the cells.

One cell allocates its PRB resources to trains in different coverage areas and tells them the corresponding limited transmitting power, which is the main idea of location and the SFR-based uplink scheduling mechanism. Assuming that one cell is divided into L regions, the PRBs from lower frequency to the higher part allocated in these area can be presented as $PRB_{1},PRB_{2},\cdots ,PRB_{L}$, respectively. When allocating resources, one cell first allocates PRB to the terminal in the area closest to the base station and then allocates resources to the next closer terminal, and finally to the farthest ones. The allocated frequency resources between two adjacent cells’ edge areas are staggered. As shown in Figure 2, cell 1 allocates $PRB_{1}$ in the $D_{1}$ area, which is close to the base station, while the same $PRB_{1}$ resource in cell 2 is allocated to the area farthest from the base station. For terminals in cell 1, the interference of data transmission on the $PRB_{1}$ frequency resource created by UEs in cell 2 can be minimized.

## 3. Problem Formulation

To reduce the negative effects that inter-cell interferences bring about, it needs not only to control the uplink transmit power, but also allocate radio resources reasonably to UE. The two problems can be formulated as the following sections.

### 3.1. Power Control of Uplink Shared Channel

The power control of the uplink shared channel has two main purposes. The first one is to adjust the transmission power on this channel to compensate negative effects caused by path loss and shadow fading; the second one is to control signal interference between cells on the same frequency.

In the traditional PUSCH (Physical Uplink Share Channel) power-control algorithm, the UE needs to set the PUSCH transmit power of subframe *i*, $P_{PUSCH}\left(i\right)$, according to the instructions from the base station:

(2) $$P_{PUSCH}\left(i\right)=\min\left\{P_{CMAX},10\log_{10}\left(M_{PUSCH}\left(i\right)\right)+P_{O\_PUSCH}\left(i\right)+\alpha \left(j\right)\cdot PL+\Delta_{TF}\left(i\right)+f\left(i\right)\right\}$$

In the above formula, $P_{CMAX}$ is the max transmit power of UE, which is defined in the protocol TS36.101. In the wireless communication environment of urban rail transit, its value is generally set to 23 dBm. $M_{PUSCH}\left(i\right)$ is the number of PRBs allocated to UE in subframe *i*; $P_{O\_PUSCH}\left(i\right)$ is the expected PUSCH transmit power, including two parts. One is the attribute parameter of cell $P_{O\_NOMINAL\_PUSCH}\left(i\right)$, and the other is the UE attribute parameter $P_{O\_UE\_PUSCH}\left(i\right)$. For the former, it is an expected power for one cell and the same for all UEs in it. However, $P_{O\_UE\_PUSCH}\left(i\right)$ is set to a different value according to different UEs.

(3) $$P_{O\_PUSCH}\left(i\right)=P_{O\_NOMINAL\_PUSCH}\left(i\right)+P_{O\_UE\_PUSCH}\left(i\right)$$

α is the compensation weight of path loss with a range 0,0.4,0.5,0.6,0.7,0.8,0.9,1. PL is the path loss on the downlink calculated by the UE. $\Delta_{TF}\left(i\right)$ indicates that different MCS modes correspond to power offsets, and $TF\left(i\right)$ is the transmission format of PUSCH; $f\left(i\right)$ is usually called transmit power control function, which belongs to a specific parameter of terminals.

As described in Section 2, the cell coverage is divided into two regions, the central and edge areas, according to the distance from the base station. Because the urban rail transit LTE-M system has a strip cell structure, this paper defines the boundary point between the central area and the edge area directly at the midpoint of the coverage radius.

In order to decide transmission power limit on the uplink, on the one hand, the received power spectral density from one terminal at base station needs to be considered; on the other hand, the maximum transmission power of the terminal needs to be considered (23 dBm in LTE-M system). It is also necessary to consider that one terminal’s uplink data transmission limit is affected from interference caused by adjacent cells. Paper [17] defines $T_{L}$ and $T_{H}$ as the minimum and the maximum interference power threshold respectively allowed in different areas of one cell. If the coverage area of the base station is divided into a central area and an edge area, the interference threshold in the two areas is $\left[T_{L}\;\;T_{H}\right]$.

Assuming that the distance between the two base stations is 1200 m and cell coverage radius is 600 m, the cell coverage area is divided into two areas with the same size. The average distance is 150 and 450 m respectively. According to the transmission loss equation of the 1.8 GHz leaky cable 4.1 dB/100m, $T_{L}$ is 0 dB and $T_{H}$ should be 4.1*(450−150)/100=12.3 dB. The threshold is $\left[0\;\;12.3\right]$ dB. If the cell is divided into four areas at equal intervals, the distance between the center point of each area and the base station is 75 m, 225 m, 375 m and 525 m from near to far, and the interference thresholds corresponding to different areas are [06.212.318.5] dB. If the cell is divided into eight areas, the interference threshold of the different areas is [03.16.29.212.315.418.521.5] dB.

Assuming that the radio resources are divided into *N* PRBs and the index of PRB is indicated by *k*, $T_{ki}$ defines the interference limit of $PRB_{k}$ on cell *i*. It supposes a specific cell has a set of neighboring cells denoted by $\left\{n\right\}$. In the environment of urban rail transit, only two neighboring cells can interfere with the uplink communication, so the number of *n* is 2. When the resource scheduler of one cell is considering assigning $PRB_{k}$ on a specific UE *q*, the serving cell could know or estimate path loss $PL\left(q,i\right)$ from UE *q* to each neighboring cell *i*, $i\in \left\{n\right\}$. For simplicity, $PL\left(q,i\right)$ is independent of *n*. Combining with the interference limit of each neighboring cell on $PRB_{k}$, the transmit power of UE *q* on this PRB, named $PI_{k}\left(q\right)$, is limited to Formula (4):

(4) $$PI_{k}\left(q\right)⩽\min_{i\in n}\left\{T_{ki}+PL\left(q,i\right)\right\}$$

### 3.2. Uplink Radio Resources Scheduling

In the above, it is assumed that PRBs are equally allocated to UEs (TAUs in trains), but the fairness between them is not considered. In addition, in an actual LTE-M system, PRB resource scheduling should consider train-wayside communication services’ requirements, channel quality, and the fairness of resource allocation among users comprehensively. Therefore, a new LTE-M uplink radio resource scheduling scheme considering the location of trains, SFR and proportional fair algorithm is proposed.

The radio resources scheduling scheme combined with proportional fair takes advantage of Max C/I (Carrier to Interference) and RR (Round Robin) methods. It is an algorithm that takes into account fairness and system capacity at the same time [18]. Formula (5) defines $m_{k,i}$, the metric of resource block *i* to the k_th UE in PF algorithm at time *j*.

(5) $$m_{k,i}=\frac{d_{k}\left(i,j\right)}{\bar{R_{k}}\left(j\right)}$$

In the formula, $d_{k}\left(i,j\right)$ is the expected instantaneous data rate obtained by the k_th UE’s resource block *i* at time *j*, which depends on the total numbers of resource blocks assigned to UE and the requirement of service data rate. $\bar{R_{k}}\left(j\right)$ is the average rate of the k_th UE in a certain time window, which is defined by Formula (6).

(6) $$\bar{R_{k}}\left(j\right)=\bar{R_{k}}\left(j-1\right)+\frac{\left[R_{k}\left(i,j\right)-\bar{R_{k}}\left(j-1\right)\right]}{T_{W}}$$

In this formula, $R_{k}\left(i,j\right)$ represents the actual instantaneous data rate of the k_th UE on the resource block *i* at time *j* and the time window parameter $T_{W}$ is taken to ensure fairness. When the PF algorithm is applied, it selects the UE with the largest PF metric and allocates resources to it.

In the requirement specification of the LTE-M system, different service rate of CBTC is specified. Under GoA (Grades of Automation) level 1/2, the service data are periodically sent by train, with a data rate above 256 kbit/s, and under GoA level 3/4, the rate requirement is 512 kbit/s. Other communication services include IMS, PIS (Passenger Information System), emergency text sending, etc. Therefore, when performing uplink service scheduling, it is necessary to ensure that the total data rate of each TAU is not less than the minimum rate required by the system. At the same time, the metric value of PF scheduling should be maximized as much as possible. The ultimate algorithmic problem description can be shown as Formulas (7) and (8):

(7) $$P:\max_{i,k}\frac{d_{k}\left(i,j\right)}{\bar{R_{k}}\left(j\right)}$$

(8) $$\begin{array}{cc} s.t.\; & R^{i}⩾r_{\min}\\ \; & p_{i}⩽p_{\max} \end{array}$$

Among them, $R^{i}$ means the total data rate, which shall not be lower than the minimum rate $r_{\min}$ specified in the requirement specification of the LTE-M system. The transmit power $p_{i}$ of one vehicular terminal cannot exceed the maximum transmit power $p_{max}$. Therefore, the radio resource scheduling and power-control problems are transformed into the above optimization ones.

## 4. Location and SFR-Based Uplink Power Control and PF Based Resources Scheduling Algorithm

To solve the above problems, an algorithm based on train locations to obtain interference tolerance thresholds in different regions of one cell is proposed in the paper. This algorithm uses the highest level of MCS (Modulation and Coding Scheme) to control the uplink transmit power. The mapping relationship between CQI (Channel Quality Index) and MCS in the LTE system is described in specification [19].

The process of uplink power control is shown in Algorithm 1. First, the base station obtains the train position information in each area of neighboring cell and the label of PRB used in each area from the neighboring through X2 interface. For example, if the cell is divided into four areas, it receives a message [0211] which is sent by the neighboring cell and means that cell coverage is divided into four areas. There are no trains in area 1, two trains in area 2, and one train each in areas 3 and 4.

In order to simplify the description of the uplink power-control algorithm, it is assumed that the number of PRBs allocated to four trains is the same. Uplink transmit of LTE system adopts a single carrier mode, which means the allocation of PRB must be continuous. We assume the sequence of allocating PRB blocks is related to the PCI (physical cell ID). If PCI is continuous, the numbers of two adjacent cells must be one odd and another even. The allocation of PRBs in one cell starts from the low-frequency band, such as $PRB_{1},PRB_{2},\cdots ,PRB_{k}$, corresponding to TAU’s distance from the base station. The opposite allocation sequence is adopted in an adjacent cell, which is $PRB_{k},PRB_{k-1},\cdots ,PRB_{1}$. In this way, the radio resources allocated in the central area of one cell is the same with PRB in the edge area of the neighboring cell. Thereby, the mutual inter-cell interference is reduced.

After receiving the above information from neighboring cells, the base station sends this to TAU through RRC (Radio Resource Control) signaling. TAU calculates the maximum transmit power PI allowed on the allocated PRB according to Equation (4). This paper assumes that power control is aiming at spectral density of a single PRB. Therefore, according (2), $M_{PUSCH}\left(i\right)$ is set to 1, and $\Delta_{TF}\left(i\right)$ and $f\left(i\right)$ are both set to 0 for simplicity. The ultimate TAU transmit power on frame *i* can be expressed as Formula (9):

(9) $$P_{PUSCH}\left(i\right)=\min\left\{\min\left\{P_{CMAX},P_{I}\right\},P_{O\_PUSCH}\left(i\right)+PL\right\}$$

In the urban rail transit data communication system, the number and magnitude of interference sources are predictable. Therefore, the SINR at the receiving end of the base station is also predictable and TAU itself can decide which MCS mechanism to use for communication.
**Algorithm 1** Location based uplink power control algorithm**Require:**Train location in neighboring cells and their PRB resource information; allocated resources in this TAU**Ensure:**TAU calculates the transmit power $P_{I}$ in according to its location and neighboring cells information1:**if** The calculated transmit power $P_{I}$ after path loss compensation is greater than the maximum transmit power $P_{CMAX}$ **then**2:   TAU selects the maximum transmit power $P_{CMAX}$ and the corresponding MCS mechanism;3:**else**4:   TAU selects the calculated transmit power $P_{I}$ and the corresponding MCS mechanism;5:**end if**6:**return** The ultimate transmit power and MCS mechanism

To ensure the fairness of different TAUs in the LTE-M system and meet the service requirements, PRB resources shall be allocated differently to TAUs in different area. When allocating RBG (Resource Block Group) for TAU, the area of the cell where the RBG is located determines the amount of resources to the TAU when neighboring cells allocate the same time-frequency resources. In order to avoid unnecessary maximum power constraints on the transmit power of neighboring cells due to RBG frequency domain misalignment in neighboring cells, some assumptions and conventions are made as following.

**Figure 3:**
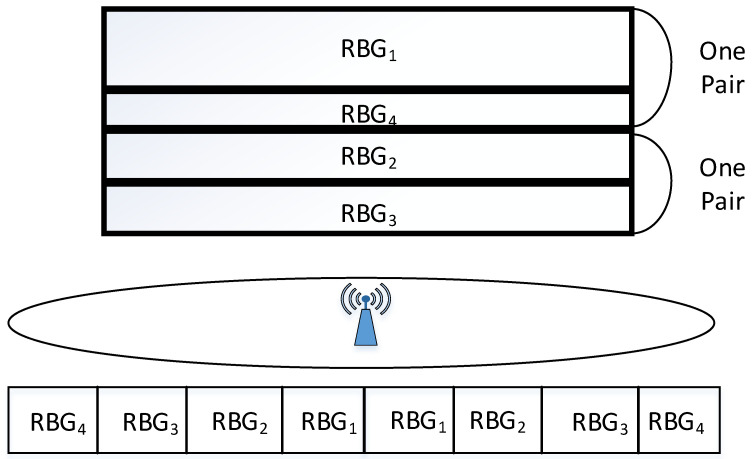
Different RBG formats in the cell.

When one cell allocates RBGs in the uplink, it combines the RBGs allocated to the TAUs in the center area and the edge area of the cell. Because the channel condition of TAU in center area is better, the number of PRBs allocated is the largest, the number of PRBs of TAU in the cell edge is the least, and the total number of PRBs of the two parts can be kept relatively stable. As shown in Figure 3, RBG1 is the radio resource allocated to users in the center of the cell, and RBG4 is the radio resource allocated to the cell edge; RBG2 is the resource allocated to users in the area close to RBG1, and RBG3 is the resource allocated to users close to RBG4. The inter-cell boundary increases the effect of inter-cell interference coordination and reduces the signaling burden.

The concrete PF-based resource scheduling algorithm design with the uplink power-control based on train position can be summarized as Algorithm 2
**Algorithm 2** Dynamic resource allocation PF algorithm with location and SFR**Require:**Train location information, coverage area *L* and bandwidth *B*, the number of PRB *N*, TAU maximum transmit power $P_{CMAX}$, and large-scale fading factor α and small-scale fading model, train CBTC service minimum transmission rate $r_{min}$ and resource availability of adjacent cells.**Ensure:**Calculate the minimum number of PRBs for each train based on the required minimum transmission rate $r_{min}$. Then the total PRBs occupied by train 1···K are marked as PRB group: $RBG_{1},RBG_{2},\cdot \cdot \cdot ,RBG_{K}$1:**if** not all trains are allocated radio resources **then**2:   According to the principle of PF, find the train $k\in \left\{1\cdot \cdot \cdot K\right\}$ and avaiable PRB $i\in \left\{1\cdot \cdot \cdot N\right\}$ that maximize the metric value $m_{k,i}=\frac{d_{k}\left(i,j\right)}{\bar{R_{k}}\left(j\right)}$;3:   According to service rate requirements $r_{min}$, the cell allocates PRB to the train *k*4:   Merge the selected PRB into the initially allocated PRB block;7:   Refresh RBG information $RBG_{k}$;6:**end if**7:**return** RBG allocation information on all trains

## 5. Simulation and Results

**Figure 4:**
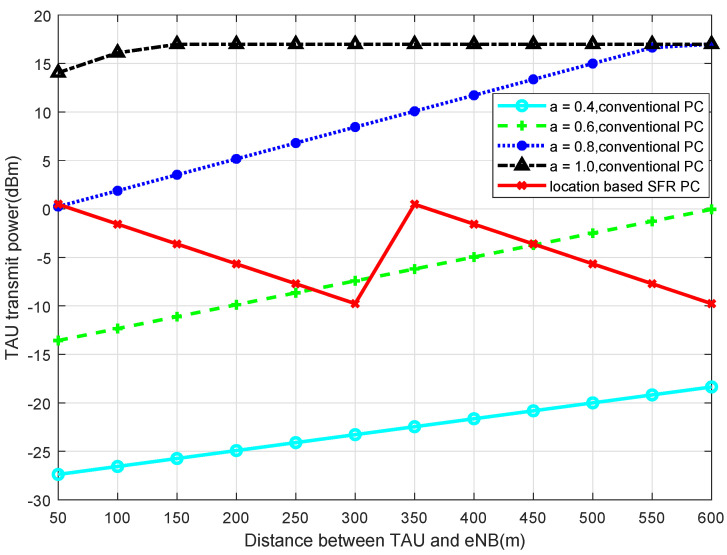
Transmit Power of TAU in 2-SFR.

**Figure 5:**
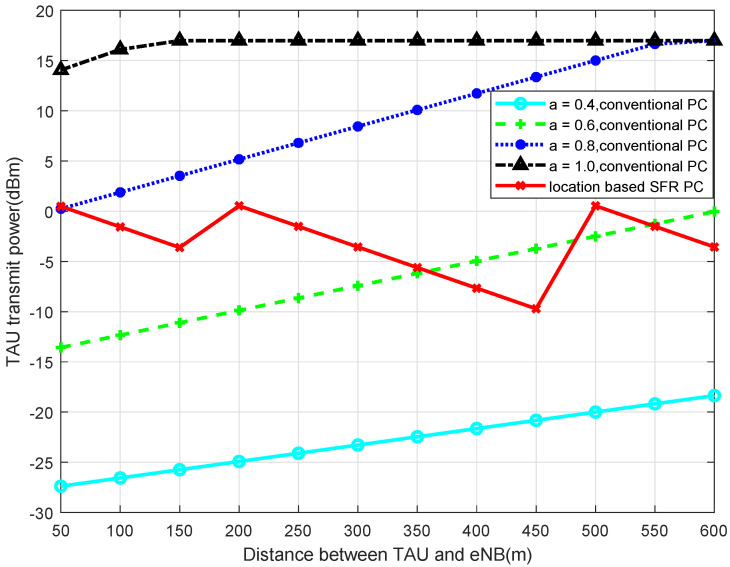
Transmit Power of TAU in 4-SFR.

**Figure 6:**
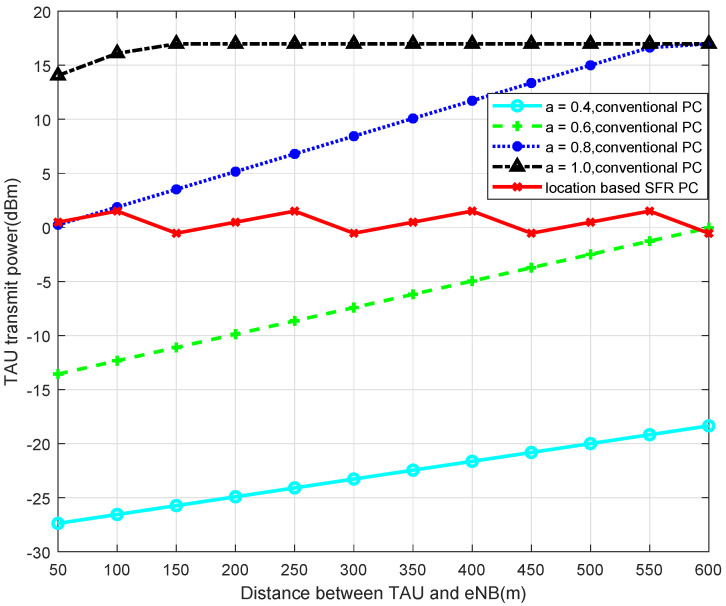
Transmit Power of TAU in 8-SFR.

**Figure 7:**
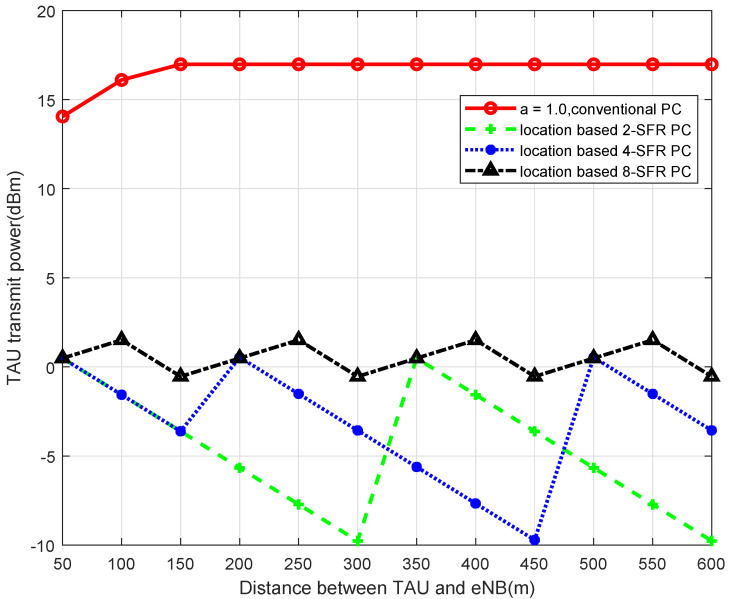
Transmit power with the traditional power control.

Simulated by MATLAB, when 2-SFR, 4-SFR, and 8-SFR with a location-based algorithm are used, the comparisons of transmit power of TAU with the traditional power-control algorithm are shown in Figure 4, Figure 5, Figure 6 and Figure 7.

It can be seen from those figures that when the power control mechanism based on location and SFR is adopted, the maximum transmit power of the UE is obviously limited, which is determined by Equation (9). And the larger the level of the SFR mechanism is, in other words, the more areas the cell is divided into, the smaller the transmission power fluctuation is. The reason is that the greater level of the SFR mechanism represents a more accurate interference threshold of each area, which can modulate TAU transmit power more accurately.

**Figure 8:**
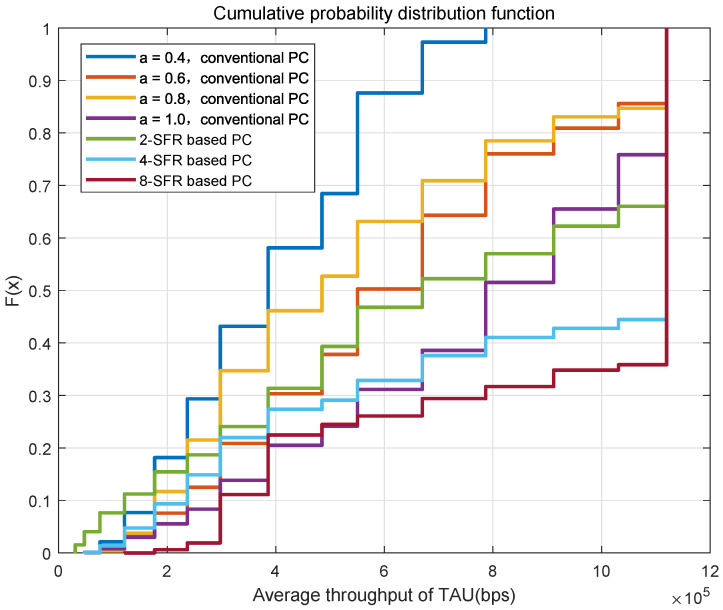
CDF of the throughput in different power control schemes.

Figure 8 shows the cumulative probability distribution function of the average throughput of all TAUs in one cell under different power-control schemes. It can be seen from the figure that the throughput of the traditional power-control scheme with α=1 is significantly better than schemes when the value is 0.4, 0.6 or 0.8. This is due to the fact that the number of trains is very small in urban rail transit, the total interference is not so serious. The throughput of the 2-SFR scheme only exceeds the traditional power control scheme with α=1 when the channel conditions are good, and the performance is weaker under bad channel conditions. Because the interference threshold in the 2-SFR power control scheme is not precise enough, the limit on the transmit power is too conservative, resulting in a clear reduction in throughput. When more SFR-level power-control schemes are used, the throughput significantly exceeds the traditional power where α is 1.

**Figure 9:**
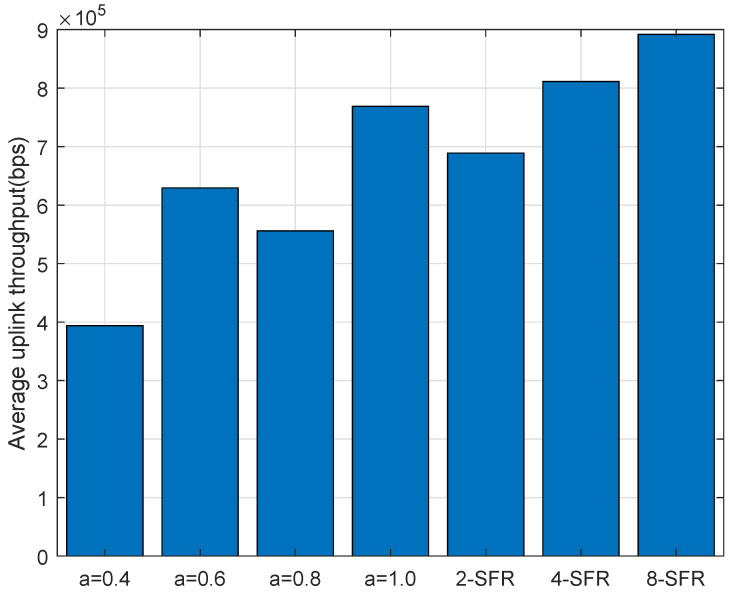
Average throughput in the uplink.

**Figure 10:**
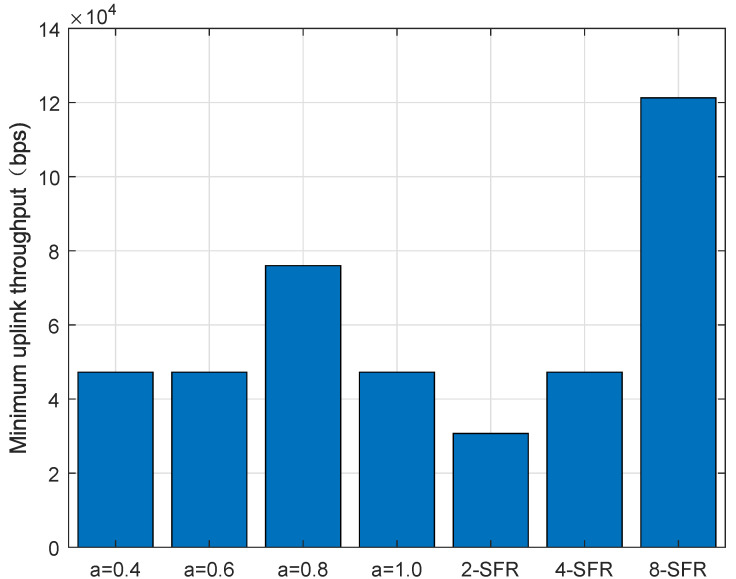
Minimum throughput in the uplink.

Figure 9 and Figure 10 are the distributions of the average uplink throughput and minimum throughput of the TAU. It can be clearly seen from the figure that 4-SFR and 8-SFR power-control schemes are better than the traditional power-control algorithm.

In order to implement the simulation of the PF-based algorithm, some important parameters are set as follows: the bandwidth of the single-cell network is 5 MHz; the cell coverage radius is 600 m; the large-scale fading model of the leaky cable is adopted; the coupling loss is 67 dB; the transmit loss is 4.1 dB/100 m; and the small-scale fading model selects the ITU-VA six-path model because of the special tunnel environment in urban rail transit [20]. Each cell coverage contains four trains with operating speeds of 60–80 km/h. The train position is updated every 200 ms, and total update time is 4000 s. The 1.8 GHz is a special frequency allocated by China’s mainland for the urban rail transit industry. The number of cells is defined as 3 with the duplex mode of TDD adopted, and the special subframe configuration is 7. All parameters are shown as Table 1.

**Figure 11:**
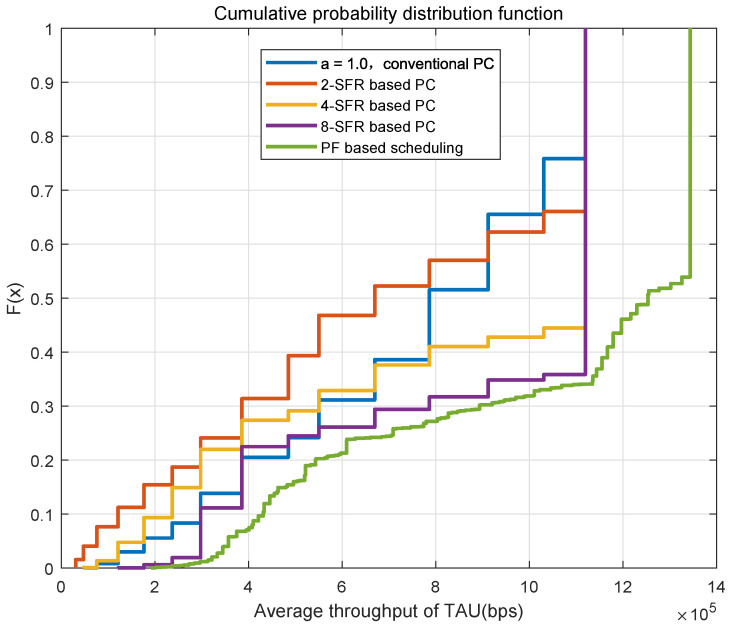
Comparison of TAU throughput between different schemes.

**Figure 12:**
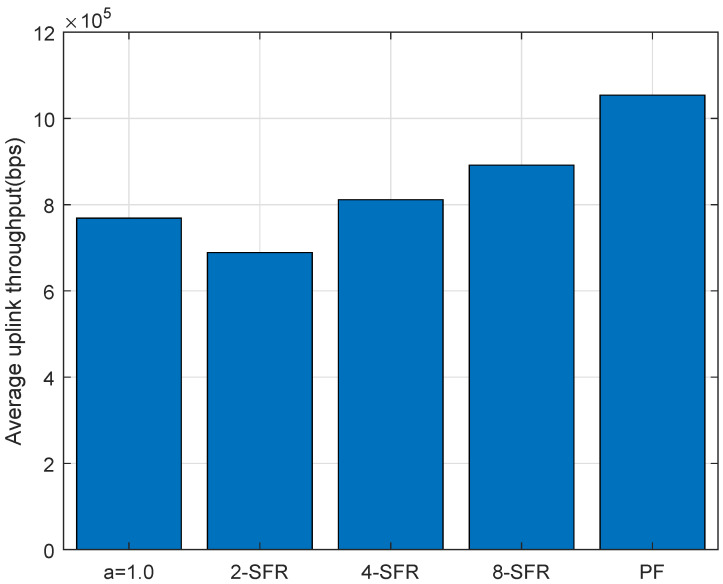
Average throughput of TAU of PF-based power control and scheduling.

Compared with complex scheduling algorithms, this algorithm has many advantages, such as simplicity and effectiveness. The TAU average throughput of the simulated PF algorithm-added scheme along with others are shown in Figure 11 and Figure 12. It can be seen from them that the total throughput of the LTE-M system has been improved after adopting the PF algorithm, and critical services’ requirements such as CBTC in urban rail transit are also met.

## 6. Conclusions

This paper studies the uplink power control and radio resource scheduling of the LTE-M system. Aiming at the characteristics of an urban rail transit vehicle-to-ground communication system for which the number of users is small and train locations are available, the uplink power-control and wireless resource scheduling scheme based on location and soft frequency reuse is designed. The simulation results show that the power-control and radio resource scheduling scheme based on the soft frequency reuse of more than four levels is better than the scheme provided by the LTE technical specification. In addition, the designed scheme requires less signaling overhead for interaction at the base station than the OI and HII interference coordination scheme defined by specifications, and it has strong practicability in the urban rail transit data communication system.

## Figures and Tables

**Table 1:** Simulation Parameters.

Parameters	Value
Frequency	1.8 GHz
Bandwidth	5 MHz
Number of cell	3
Radius of eNodeB coverage	600 m
Duplexision method	TDD
Uplink–downlink subframe configuration	1
Special subframe configuration	7
Path loss model	67+4.1×d/100
Small scale loss model	ITU-VA six paths model
The max transmitting power of eNodeB	43 dBm
Density of noise frequency	−174 dBm/Hz
The max transit power of TAU	23 dBm
The min service data rate	256 Kbps
Total number of PRB	25
Block Error Rate	$10^{-3}$
Layer numbers of SFR	2, 4, 8
Speed of train	60∼80 km/h

## Abbreviations

The following abbreviations are used in this paper:

LTE	Long Term Evolution
LTE-M	Long Term Evolution for Metro
CBTC	Communication Based Train Control
TAU	Train Access Unit
ICI	Inter-Cell Interference
PL	Path Loss
FFR	Fractional Frequency Reuse
SFR	Soft Frequency Reuse
OI	Overload Indicator
HII	High Interference Indicator
